# CAR-T cell therapy: current limitations and potential strategies

**DOI:** 10.1038/s41408-021-00459-7

**Published:** 2021-04-06

**Authors:** Robert C. Sterner, Rosalie M. Sterner

**Affiliations:** 1 Medical Scientist Training Program, University of Wisconsin-Madison, School of Medicine and Public Health, Madison, WI USA; 2 Department of Surgery, Mayo Clinic, Rochester, MN USA

**Keywords:** Immunotherapy, Cancer immunotherapy

## Abstract

Chimeric antigen receptor (CAR)-T cell therapy is a revolutionary new pillar in cancer treatment. Although treatment with CAR-T cells has produced remarkable clinical responses with certain subsets of B cell leukemia or lymphoma, many challenges limit the therapeutic efficacy of CAR-T cells in solid tumors and hematological malignancies. Barriers to effective CAR-T cell therapy include severe life-threatening toxicities, modest anti-tumor activity, antigen escape, restricted trafficking, and limited tumor infiltration. In addition, the host and tumor microenvironment interactions with CAR-T cells critically alter CAR-T cell function. Furthermore, a complex workforce is required to develop and implement these treatments. In order to overcome these significant challenges, innovative strategies and approaches to engineer more powerful CAR-T cells with improved anti-tumor activity and decreased toxicity are necessary. In this review, we discuss recent innovations in CAR-T cell engineering to improve clinical efficacy in both hematological malignancy and solid tumors and strategies to overcome limitations of CAR-T cell therapy in both hematological malignancy and solid tumors.

## Introduction

**Fig. 1:**
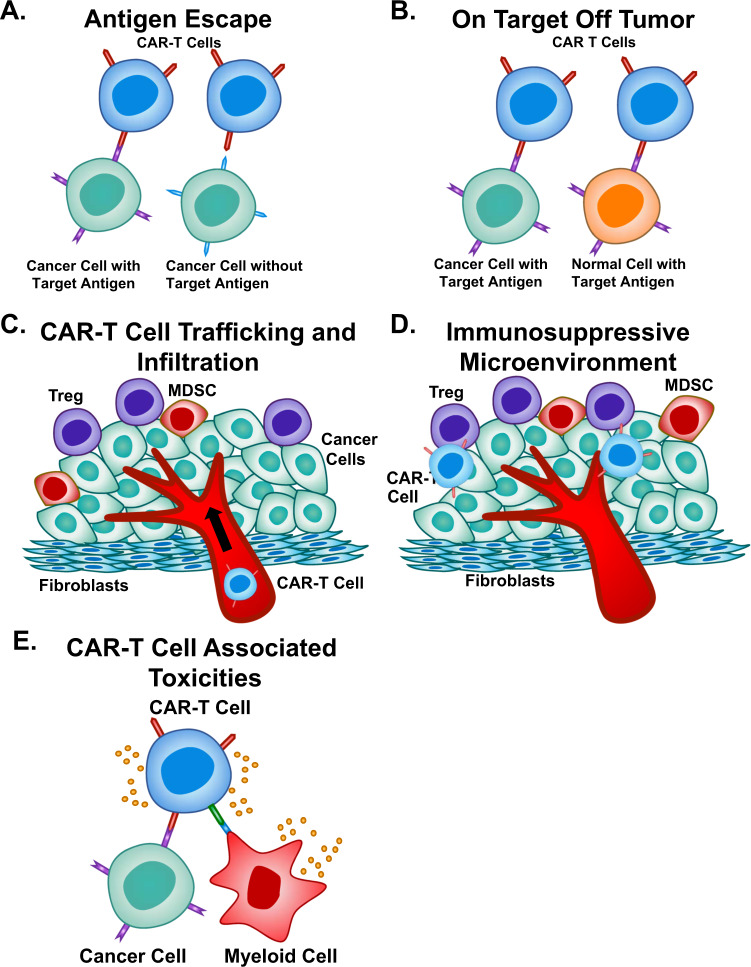
Limitations of CAR-T Cell Therapy. Current challenges in CAR-T cell therapy include (**A**) antigen escape, (**B**) on-target off-tumor effects, (**C**) trafficking and infiltration of tumors, (**D**) the immunosuppressive tumor microenvironment, and (**E**) CAR-T cell-associated toxicities.

Chimeric antigen receptor (CAR)-T cell therapy has been revolutionary as it has produced remarkably effective and durable clinical responses^1^. CARs are engineered synthetic receptors that function to redirect lymphocytes, most commonly T cells, to recognize and eliminate cells expressing a specific target antigen. CAR binding to target antigens expressed on the cell surface is independent from the MHC receptor resulting in vigorous T cell activation and powerful anti-tumor responses^2^. The unprecedented success of anti-CD19 CAR-T cell therapy against B cell malignancies resulted in its approval by the US Food and Drug Administration (FDA) in 2017^3–5^. However, there are major limitations to CAR-T cell therapy that still must be addressed including life-threatening CAR-T cell-associated toxicities, limited efficacy against solid tumors, inhibition and resistance in B cell malignancies, antigen escape, limited persistence, poor trafficking and tumor infiltration, and the immunosuppressive microenvironment. In addition, the workforce must adapt to meet the needs of this growing and evolving field by developing educational programs to train a workforce^6^. Many approaches including combining CAR-T cell therapy with other anticancer therapies or employing innovative CAR engineering strategies to improve anti-tumor efficacy, expand clinical efficacy, and limit toxicities have been proposed. In this review, we discuss recent innovations in CAR-T cell engineering to improve clinical efficacy in both hematological malignancy and solid tumors and strategies to overcome current limitations (Table 1), including antigen escape, CAR-T cell trafficking, tumor infiltration, the immunosuppressive microenvironment, and CAR-T cell-associated toxicities (Fig. 1).

**Table 1:** CAR-T cell therapy current limitations and potential strategies.

Limitations of CAR-T cell therapy	Potential strategies
Antigen escape	Targeting multiple antigens (dual or tandem CARs) **∙**Preliminary clinical trial results of CD19/CD22 targeted CARs for treatment of ALL/DLBCL and CD19/BCMA targeted for multiple myeloma have demonstrated promising efficacy^48–51^. **∙**Solid tumor: HER2 /IL13Ra2 (glioblastoma) and HER2/MUC1 (breast cancer) CARs produce superior antitumor responses compared to single target therapy^28,52^.
On-target off-tumor effects	Targeting tumor-restricted post-translational modifications **∙**Four major CAR-T cell targets have been investigated: TAG72^28^, B7-H3^55,56^, MUC1^16^, and MUC16^57,58^.
CAR-T cell trafficking and tumor infiltration	Local administration vs systemic delivery **∙**Superior therapeutic efficacy of intrapleural^63^ and intraventricular^61,62^ injection of CAR-T cells in mesothelioma and glioblastoma/brain cancer patients, respectively. Expressing chemokine receptors on CAR-T cells that match and respond to tumor-derived chemokines **∙**Integrin αvβ6-CAR-T cells modified to express CXCR2 or CAR-T cells overexpressing CXCR1/CXCR2 enhance trafficking and significantly improve antitumor efficacy^64–66^. Engineering CAR-T cells to enhance penetration through physical barriers (tumor stroma) **∙**CAR-T cells that express heparanase or fibroblast activation protein targeted CAR-T cells have shown enhanced infiltration and antitumor activity^68,69^.
Immunosuppressive microenvironment	Combination immunotherapy with CAR-T cells and checkpoint blockade **∙**In hematological malignancy, combination PD-1 blockade and CD19 CAR-T cell therapy in B-ALL patients improved outcomes and improved CAR-T cell persistence^73^. **∙**In solid tumors, many studies are currently evaluating combination therapy^71,74^. Engineering CAR-T cells to provide immunostimulatory signals in the form of cytokines or CARs resistant to immunosuppressive factors. **∙**Engineering CARs to provide immunostimulatory signals have relied on IL-12 secretion^78^, IL-15 expression^79^, and redirecting immunosuppressive cytokines (e.g., IL-4) resulting in increased survival, proliferation, and antitumor activity^80^. **∙**CARs resistant to immunosuppressive factors in the hostile tumor microenvironment such as TGF β-mediated inhibitory signals have been developed^76^.
CAR-T cell-associated toxicities	Altering CAR structure to ameliorate toxicity **∙**Decreasing CAR antigen-binding domain affinity to micromolar affinity^9^. **∙**Cytokine secretion can be modulated by modifying the CAR hinge and transmembrane regions^93^. **∙**Tailoring the costimulatory domain based on tumor type, tumor burden, antigen density, target antigen–antigen binding domain pair, and concerns of toxicity^94^. **∙**CAR immunogenicity can be decreased by utilizing human/humanized antibody fragments instead of murine-derived CARs^25,95,96^. Modifying CAR transduced T cells and neurotoxicity **∙**Inhibition of macrophage activating and monocyte activating cytokine GM-CSF with lenzilumab decreases CRS and neurotoxicity and increases CAR-T cell activity^87,98,99^. **∙**IL-1 receptor antagonists reduce a form of neuroinflammation in leukemia/lymphoma mouse models^102^. CAR “off-switches”. **∙**CAR constructs engineered to express CD20 facilitate depletion of CAR-T cells via rituximab treatment^104^. **∙**Dasatinib treatment has exciting potential as it provides temporary inhibition of CAR-T cell function and could allow for rescue therapy after toxicities have subsided^107^.

Summary of major limitations of CAR-T cell therapy and potential strategies to overcome limitations.

## CAR Structure

CARs are modular synthetic receptors that consist of four main components: (1) an extracellular target antigen-binding domain, (2) a hinge region, (3) a transmembrane domain, and (4) one or more intracellular signaling domains. Here we will discuss the current principles underlying CAR design.

### Antigen binding domain

The antigen binding domain is the portion of the CAR that confers target antigen specificity. Historically, the antigen-binding domains are derived from the variable heavy (V_H_) and light (V_L_) chains of monoclonal antibodies, connected via a flexible linker to form a single-chain variable fragment (scFv). Classically, the scFvs present in CARs target extracellular surface cancer antigens resulting in major histocompatibility complex (MHC)-independent T cell activation, although recognition of intracellular tumor-associated antigens using MHC-dependent, T cell receptor (TCR)-mimic CARs have been described^7^. Several characteristics of the scFv impact CAR function beyond simply recognizing and binding the target epitope. For instance, the mode of interaction among the V_H_ and V_L_ chains as well as the complementarity-determining regions’ relative positions impact the affinity and specificity of the CAR for its target epitope^8^. Affinity is a particularly important antigen-binding domain parameter as it fundamentally determines CAR function. In order to recognize antigens on tumor cells, induce CAR signaling, and activate T cells, the CARs antigen binding affinity must be sufficiently high but not high enough to result in activation induced death of the CAR expressing T cell and trigger toxicities (discussed later in this review)^9,10^. While affinity is certainly one of the most important factors to further complicate matters, it has been shown that even scFvs with similar affinities can differentially impact CAR-T cell function. Therefore, in order to optimize binding of the CAR to its target antigen, additional factors such as epitope location, target antigen density, and avoidance of scFvs associated with ligand-independent tonic signaling must be considered.

### Hinge region

The hinge or spacer region is defined as the extracellular structural region that extends the binding units from the transmembrane domain. The hinge functions to provide flexibility to overcome steric hindrance and contributes to the length in order to allow the antigen-binding domain to access the targeted epitope. Importantly, the selected hinge appears to impact CAR functionality as differences in the length and composition of the hinge region can affect flexibility, CAR expression, signaling, epitope recognition, strength of activation outputs, and epitope recognition^11,12^. In addition to these affects, it has been proposed that the spacer length is critical to provide sufficient intercellular distance to allow for immunological synapse formation^13^. In principle, the “optimal” spacer length is dependent on the position of the target epitope and the level of steric hindrance on the target cell in which long spacers provide added flexibility and allow more effective access to membrane-proximal epitopes or complex glycosylated antigens, while short hinges are more successful at binding membrane-distal epitopes^11,14–16^. In practice, however, the proper spacer length is often determined empirically and must be tailored for each specific antigen-binding domain pair. There are numerous examples in the literature of short spacer CARs (CD19 and carcinoembryonic antigen (CEA))^14^ and long spacer CARs (mucin 1 (MUC1)), membrane-proximal epitopes of receptor tyrosine kinase-like orphan receptor 1 (ROR1)^16^. The most commonly employed hinge regions are derived from amino acid sequences from CD8, CD28, IgG1, or IgG4. IgG-derived spacers, however, can cause CAR-T cell depletion and thus, decreased persistence in vivo as they can interact with Fcγ receptors^17,18^. These effects can be avoided by either the selection of a different spacer region or through additional engineering of the spacer region based on functional or structural considerations.

### Transmembrane domain

Among all of the components of CAR’s, the transmembrane domain is probably the least characterized region. The major function of the transmembrane domain is to anchor the CAR to the T cell membrane, although evidence suggests that the transmembrane domain can also be relevant for CAR-T cell function^19,20^. More specifically, studies suggest that the CAR transmembrane domains influence CAR expression level, stability, can be active in signaling or synapse formation, and dimerize with endogenous signaling molecules^19–21^. Most transmembrane domains are derived from natural proteins including CD3*ζ*, CD4, CD8α, or CD28. The effect of one transmembrane compared to another on CAR function is not well studied as the transmembrane domain is frequently changed based on the requirements of the extracellular spacer region or the intracellular signaling domains. Notably, the CD3*ζ* transmembrane may facilitate CAR-mediated T cell activation as the CD3*ζ* transmembrane domain mediates CAR dimerization and incorporation into endogenous TCRs^19^. These beneficial effects of the CD3*ζ* transmembrane domain come at the cost of decreasing CAR stability compared to CAR’s with the CD28 transmembrane domain^22^. Together, the impact of the transmembrane domain and the hinge region appear to also influence CAR-T cell cytokine production and activation induced cell death (AICD) as CAR-T cells with CD8α transmembrane and hinge domains release decreased amounts of TNF and IFNγ and have decreased susceptibility to AICD compared to CARs with these domains derived from CD28^23^. Overall, studies suggest that proper CAR-T cell signaling may be best facilitated by linking the proximal intracellular domain to the corresponding transmembrane domain, while CAR expression and stability may be enhanced by using the frequently used CD8α or CD28 transmembrane domains.

### Intracellular signaling domain(s)

Arguably the most attention in CAR engineering has been focused on understanding the effects of CAR co-stimulation with the goal of generating CAR constructs with the optimal endodomain. First generation CARs engineered in the late 1990s contained a CD3*ζ* or FcRγ signaling domain^24^. A large majority of CARs rely on activation of CAR-T cells through CD3*ζ* derived immunoreceptor tyrosine-based activation motifs^25^. Effective T cell responses are not able to be generated by only signaling with these motifs however^26^. The durability and persistence of these first generation CARs are not robust in vitro^26^. These findings were echoed by clinical studies that showed limited or no efficacy^27,28^.

The importance of co-stimulation in CD-19-targeted CAR-T cell persistence was demonstrated using early in vivo models of B-cell malignancies^29^. IL-2 production and proliferation upon repeated antigen exposure were improved by adding a co-stimulatory domain^30^. With this understanding of the importance of co-stimulation for durable CAR-T cell therapy, second generation CARs with one co-stimulatory domain in series with the CD3*ζ* intracellular signaling domain were generated^30,31^. The two most common, FDA-approved co-stimulatory domains CD28 and 4-1BB (CD137) are both associated with high patient response rates. The co-stimulatory domains differ in their functional and metabolic profiles in which CARs with CD28 domains differentiate into effector memory T cells and primarily use aerobic glycolysis while CARs possessing the 4-1BB domain differentiate into central memory T cells and display increased mitochondrial biogenesis and oxidative metabolism^32^. Clinically, second generation CAR-T cells have produced strong therapeutic responses in several hematological malignancies, including chronic lymphocytic leukemia, B-cell acute lymphoblastic leukemia, diffuse large B-cell lymphoma, and multiple myeloma and the efficacy of second generation CAR-T cells are currently being investigated in solid tumors, including glioblastoma, advanced sarcoma, liver metastases, as well as mesothelioma, ovarian cancer, and pancreatic cancer^33^. Several alternative co-stimulatory domains such as inducible T cell co-stimulator (ICOS)^34^, CD27 (ref. ^35^), MYD88 and CD40 (ref. ^36^), and OX40 (CD134) (ref. ^37^) have demonstrated preclinical efficacy although clinical investigation is still pending. It has been hypothesized that co-stimulation through only one domain produces incomplete activation, resulting in the production of third generation CARs, which incorporate two costimulatory domains in series with CD3*ζ*^38^. Preclinical studies of third generation CARs have produced mixed results. Specifically, CARs incorporating CD28 and 4-1BB signaling resulted in stronger cytokine production in lymphoma, and pulmonary metastasis showed an improved in vivo antitumor response compared to second generation CARs^39^. In leukemia and pancreatic cancer models, third generation CARs showed no in vivo treatment benefits and failed to outperform second generation CARs in their respective models^40,41^.

## Limitations of CAR-T cell therapy

### Antigen escape

One of the most challenging limitations of CAR-T cell therapy is the development of tumor resistance to single antigen targeting CAR constructs. Although initially single antigen targeting CAR-T cells can deliver high response rates, the malignant cells of a significant portion of patients treated with these CAR-T cells display either partial or complete loss of target antigen expression. This phenomenon is known as antigen escape. For example, although 70–90% of relapsed and/or refractory ALL patients show durable responses to CD19 targeted CAR-T cell therapy, recent follow-up data suggest development of a common disease resistance mechanism, including downregulation/loss of CD19 antigen in 30–70% of patients who have recurrent disease after treatment^42,43^. Similarly, downregulation or loss of BCMA expression in multiple myeloma patients being treated with BCM targeted CAR-T cells has been observed^44–46^. Similar antigen escape resistance patterns have been observed in solid tumors. For example, a CAR-T cell therapy case report that targeted IL13Ra2 in glioblastoma suggested that tumor recurrences displayed decreased IL13Ra2 expression^47^. In order to reduce the relapse rate in CAR-T cell treatment of both hematological malignancies and solid tumors, many strategies are now relying on targeting multiple antigens. These employ the use of either dual CAR constructs or tandem CARs, which is a single CAR construct that contains two scFvs in order to concomitantly target multiple target tumor antigens. Clinically, it appears that both of these strategies may result in prolonged durable remission rates, and there are several CD19 and CD20 or CD 19 and CD22 clinical trials^25^. Excitingly, preliminary results from clinical trials using dual-targeted CAR-T cells (CD19/CD22 or CD19/BCMA) have demonstrated promising results^48–51^. More specifically, preliminary clinical trial results of CD19/CD22 CAR-T cell therapy have demonstrated promising efficacy in adult patients with ALL and diffuse large B cell lymphoma^50,51^. Furthermore, preliminary results of BCMA/CD19 targeted CARs in the treatment of multiple myeloma suggest BCMA/CD19 targeted CARs are highly efficacious with favorable safety profiles^48,49^. In solid tumors, several tandem CARs have been tested in preclinical models including HER2 and IL13Ra2 in glioblastoma and HER2 and MUC1 in breast cancer. In both cases, dual targeting resulted in superior anti-tumor responses compared to single targeted therapy^28,52^. In the glioblastoma study, CARs targeting HER2 and IL13Ra2 led to improved anti-tumor activity and decreased antigen escape when compared against two other dual-targeting therapies^53^. This study illustrates the importance of optimizing the selection of target antigens that not only improve antitumor response but also decrease antigen escape mechanisms to prevent relapse.

### On-target off-tumor effects

One of the challenges in targeting solid tumor antigens is that solid tumor antigens are often also expressed on normal tissues at varying levels. Therefore, antigen selection is crucial in CAR design to not only ensure therapeutic efficacy but also to limit “on-target off-tumor” toxicity. A potential avenue to overcome the targeting of antigens on solid tumors that are also present on normal tissues is the targeting of tumor-restricted post-translational modifications such as solid tumor overexpressed truncated O-glycans such as Tn (GalNAca1-O-Ser/Thr) and sialyl-Tn (STn) (NeuAca2–6-GalNAca1-O-Ser/Thr)^54^. Four major CAR-T cell targets have been investigated including TAG72^28^, B7-H3 (refs. ^55,56^), MUC1 (ref. ^16^), and MUC16 (refs. ^57,58^). Although first generation CAR-T cells targeting TAG72 in colorectal cancer produced no anti-tumor response, new versions of second generation TAG72-CAR-T cells and other tumor-restricted post-translational modifications are currently being investigated^28,59^. Further development of innovative strategies to reduce antigen escape and select antigens capable of inducing a sufficient antitumor efficacy, while minimizing toxicity concerns will be necessary in order to expand the clinical use of CAR-T cell therapies in hematological malignancy and solid tumors.

### CAR-T cell trafficking and tumor infiltration

Compared to hematological malignancies, solid tumor CAR-T cell therapy is limited by the ability of CAR-T cells to traffic to and infiltrate solid tumors as the immunosuppressive tumor microenvironment and physical tumor barriers such as the tumor stroma limit the penetration and mobility of CAR-T cells. One strategy to ameliorate these limitations is through the utilization of delivery routes other than systemic delivery as local administration (1) eliminates the need for CAR-T cells to traffic to disease sites and (2) limits on-target off-tumor toxicities as the CAR-T cells’ on-target activity is directed on tumor cells minimizing interaction with normal tissues^60^. Preclinical models have demonstrated superior therapeutic efficacy of intraventricular injection of CAR-T cells targeting HER2 (ref. ^61^) and IL13Ra2 (ref. ^62^) in breast cancer brain metastases and in glioblastoma. These studies have led to three ongoing clinical trials investigating intraventricular injection of CAR-T cells in glioblastoma (NCT02208362, NCT03389230) and recurrent brain or leptomeningeal metastases (NCT03696030). Similarly, preclinical models showed superior CAR-T cell treatment of malignant pleural mesothelioma through intrapleural injection, which has resulted in an ongoing phase 1 clinical trial (NCT02414269) (ref. ^63^). Although localized injection appears to have superior efficacy, theoretically this approach is limited to single tumor lesions/oligometastatic disease^25^.

One recently developed strategy that appears to significantly improve CAR-T cell trafficking involves expressing chemokine receptors on CAR-T cells that match and respond to tumor-derived chemokines^64^. For example, recent studies have demonstrated that integrin αvβ6-CAR-T cells modified to express CXCR2 or CAR-T cells overexpressing CXCR1 or CXCR2 both enhance trafficking and significantly improve antitumor efficacy^64–66^. Physical barriers such as the tumor stroma also limit CAR-T cell therapy as these physical barriers prevent tumor penetration. Stroma is mostly composed of extracellular matrix in which heparin sulfate proteoglycan (HSPG) is the primary component that CAR-T cells must degrade in order to pass into the tumor^67^. CAR-T cells that have been engineered to express heparanase, an enzyme that degrades HSPG, show enhanced tumor infiltration and antitumor activity^68^. Similarly, fibroblast activation protein (FAP)-targeted CAR-T cells demonstrate increased cytotoxic function through reducing tumor fibroblasts in animal models^69^. In the future, there is a need for the development of innovative delivery strategies and approaches to improve tumor penetration in order to extend therapeutic efficacy to complex solid tumors and metastases.

### Immunosuppressive microenvironment

In the tumor microenvironment, many cell types that drive immunosuppression can infiltrate solid tumors including myeloid-derived suppressor cells (MDSCs), tumor-associated macrophages (TAMs), and regulatory T cells (Tregs)^70^. These infiltrates and tumor cells drive the production of tumor facilitating cytokines, chemokines, and growth factors. In addition, immune checkpoint pathways such as PD-1 or CTLA-4 can serve to decrease antitumor immunity. One of the main causes of no response or a weak response to CAR-T cell therapy is poor T cell expansion and short-term T cell persistence. It has been hypothesized that development of this T cell exhaustion is triggered by co-inhibitory pathways^71^. Therefore, combination immunotherapy with CAR-T cells and checkpoint blockade is thought to be the next immunotherapy frontier as it provides the two elements necessary for strong immune responses:^1^ CAR-T cells, which provide the infiltrate and^2^ PD-1/PD-L1 blockade, which can ensure sustained T cell persistence and function^72^. In hematological malignancy, combination PD-1 blockade and CD19 CAR-T cell therapy in 14 children with heavily pretreated B-ALL resulted in improved persistence of CAR-T cells and better outcomes at a single-center study at Children’s Hospital of Pennsylvania^73^. In solid tumors, there are currently many studies aiming to evaluate the response rate of combination therapy^71,74^. One intriguing study in which 11 mesothelioma patients who received preconditioning with cyclophosphamide followed by a single dose of mesothelin targeted CAR-T cells and at least three doses of anti-PD-1agent resulted in a 72% response rate and complete metabolic responses in two patients^75^. Combining other forms of immunotherapy strategies may still be necessary in order to combat the inhibitory signal present in the tumor microenvironment.

Recently efforts have focused on engineering CARs that are resistant to immunosuppressive factors in the hostile tumor microenvironment such as TGF β-mediated inhibitory signals^76^. Another intriguing strategy involves the engineering of CAR-T cells to provide immunostimulatory signals in the form of stimulatory cytokines that increase survival, proliferation, antitumor activity of T cells, and rebalance the tumor microenvironment^77^. Many studies have investigated numerous cytokines to create these “armored CARs”. Studies that focus on the expression of pro-inflammatory cytokines instead of focusing on inhibitory signals have relied on IL-12 secretion^78^, IL-15 expression^79^, and redirecting immunosuppressive cytokines (e.g., IL-4) signaling towards proinflammatory cytokines^80^.

Although combination checkpoint blockade-CAR-T cell therapy is likely a new immunotherapy option, it is important to also recognize that even this combination may still be insufficient to induce infiltration of T cells and effector function. Therefore, additional studies combining CAR-T cell therapy and checkpoint blockade with other immunotherapies/strategies may be necessary to result in T cell infiltration and effector function in complex hematological malignancies or solid tumors.

### CAR-T cell-associated toxicities

Although CAR-T cell therapy has been a revolutionary cancer treatment tool, high rates of toxicities with some fatalities have prevented CAR-T cell therapy from becoming first-line treatment. Critical factors that likely determine the incidence and severity of CRS, HLH/MAS, and/or ICANS are the design of the CAR, the specific target, and the tumor type^81^. To date, the toxicities underlying CAR-T cell therapy have been most extensively characterized in patients receiving the first FDA approved CAR-T cell therapy, CD19-directed CARs^82,83^. Even in the clinical trials with the most dramatic response rates, severe, life-threatening events have occurred in patients^4,5,84^. Specifically, in the case of acute lymphoblastic leukemia/lymphoma (ALL/LBL) patients treated with CAR-T cell therapy, nearly all patients have at least some less severe toxicity manifestations while 23–46% of patients displayed severe supraphysiologic cytokine production and massive in-vivo T cell expansion^85^. These toxic levels of systemic cytokine release and severe immune cell cross-activation in some patients result in the following toxicities:^1^ cytokine-release syndrome (CRS), which is associated with supraphysiologic cytokine production and massive in vivo T cell expansion^2^ hemophagocytic lymphohistiocytosis and/or macrophage activation syndrome (MAS) defined as a severe hyperinflammatory syndrome characterized by CRS and combinations of elevated serum ferritin and hemophagocytosis, renal failure, liver enzymes, splenomegaly, pulmonary edema, and/or absence of NK cell activity, and^3^ immune effector cell-associated neurotoxicity syndrome (ICANS), which is characterized by elevated cerebrospinal fluid cytokine levels and blood–brain barrier disruption^86^.

Mechanistically, CRS is a result of administered CAR-T cells becoming extensively activated resulting in the release of massive amounts of cytokines. Clinical manifestations of mild CRS is fever accompanied by fatigue, diarrhea, headache, rashes, arthralgia, and myalgia and in more severe cases, patients may present with hypotension, cardiac dysfunction, circulatory collapse, respiratory failure, renal failure, multiorgan system failure, and with possible progression to death^3,4,87^. In total, 77–93% of patients with leukemia receiving CAR-T cell therapy and 37–93% of patients with lymphoma receiving CAR-T cell therapy had any grade of CRS while 46% of patients treated with tisagenlecleucel for relapsed/refractory B-ALL and 13–18% of patients treated with axicabtagene ciloleucel and tisagenlecleucel, respectively for diffuse large B-cell lymphoma had ≥Grade 3 CRS^3,4^. Pathophysiologically, CRS is believed to be primarily mediated by IL-6 and therefore, management relies on the use of IL-6 receptor blockade with tocilizumab and corticosteroids^3–5^. Even with the use of tocilizumab, which is FDA approved to treat severe CRS, severe CRS and death still occur. Interestingly, HLH/MAS secondary to CAR-T cell therapy can be refractory to IL-6 inhibition and instead may require chemotherapy. While the incidence of HLH/MAS secondary to CAR-T cell therapy is unclear due to overlap with high-grade CRS, it has been reported in ≈1% of patients receiving CAR-T cell therapy^88^. In the case of neurotoxicity, the underlying pathophysiology and mechanisms are not completely understood^88^. Clinical manifestations of ICANS range from confusion, headache, attention deficits, word-finding difficulties, focal neurological deficits, or encephalopathy to life-threatening cerebral edema, transient coma, or seizures^89^. Neurotoxicity following CAR-T cell therapy is relatively common and can occur in up to 67% and 62% of patients receiving treatment for leukemia and lymphoma, respectively^86^. Management of neurotoxicity focuses on corticosteroids as IL-6 inhibitors are often not effective for neurotoxicity associated with CAR-T cell therapy^90,91^. To date, there remain no approved therapies for the prevention of the above toxicities, making it essential to optimize CAR engineering and employ other strategies to decrease CAR-induced toxicities^88^. Below, we review lessons learned in engineering CARs to reduce toxicity and additional strategies to ameliorate toxicities in CAR-T cell therapy.

### Engineering CAR-T cells to ameliorate toxicity

In order to achieve efficacious therapeutic responses, a CAR-T cell antigen-binding domain must bind its target epitope and reach a minimum threshold level to induce CAR-T cell activation and cytokine secretion. At the same time, however, there is also some threshold level of activation that when surpassed produces toxic levels of cytokines and immune system activation. In other words, the CAR-T cell must remain within its therapeutic window to be clinically effective as overshooting the therapeutic window will lead to toxicity. From an engineering perspective, the degree of CAR-T cell activation and activation kinetics are influenced by several factors including but not limited to the level of tumor antigen expressed on malignant cells, tumor burden, antigen binding domain’s affinity to its target epitope, and the CAR’s costimulatory elements^33,92^. Therefore, careful consideration of several components of the CAR’s modular structure is necessary to optimize therapeutic efficacy and limit toxicity.

### Altering CAR structure

One route to decrease toxicity is through altering the affinity of the CAR-T cell’s antigen binding domain. Decreasing the affinity of the antigen-binding domain would be expected to result in an increased requirement for higher antigen density on tumor cells in order for high levels of activation to be achieved. Therefore, it would be expected that decreased antigen affinity would circumvent the targeting of healthy tissue with a relatively low amount of antigen. Studies investigating this rationale have demonstrated that antigen-binding domains with micromolar affinity were much more selective for tumors with higher levels of target antigen expression compared to antigen binding domains with low nanomolar/sub-nanomolar affinity^9^.

It is also possible to modulate cytokine secretion via activated CAR-T cells by modifying the hinge and transmembrane regions. For instance, in a CD19-targeted CAR, modification of the CD8-α derived hinge and transmembrane amino acid sequences led to lower levels of cytokine release and decreased CAR-T cell proliferation^93^. Optimizing the hinge and transmembrane regions could be a useful approach to decrease toxicity as in phase 1 clinical trial, these modified hinge and transmembrane region CARs resulted in complete remission in 54.5% of B cell lymphoma patients (6/11 patients), and importantly, there were no CRS or ICANS events grade >1^93^.

The costimulatory domain offers another modifiable region in CAR design that can be tailored based on tumor type, tumor burden, antigen density, target antigen–antigen binding domain pair, and concerns of toxicity. Specifically, 4-1BB domains result in a lower risk of toxicities, higher T cell endurance, and a lower peak level of T cell expansion, while CD28 co-stimulatory domains are associated with CAR-T cell activity that is more rapid in onset and subsequent exhaustion^94^. Therefore, 4-1BB costimulatory domains, which produce less toxicity may be particularly useful in cases where there is a high disease burden and/or a high antigen density tumor, while CD28 costimulatory domains may be necessary in order to achieve the required T cell activation threshold in cases where there is low total surface antigen density and/or a low-affinity antigen binding domain CAR^94^.

### CAR Immunogenicity

The recognition of CAR constructs by the host immune system may contribute to cytokine-related toxicities and thus, utilizing human or humanized antibody fragments instead of murine-derived CARs to decrease CAR immunogenicity may be advantageous^25,95^. In addition, the hinge and/or transmembrane domains can be modified in order to decrease the immunogenicity of CAR, and also interestingly CAR-T cell persistence is improved^95,96^.

### Modifying CAR transduced T cells and neurotoxicity

An exciting, recently developed avenue to prevent CAR-T cell cytokine toxicities is based on modifying the CAR transduced T cells. Cytokines and myeloid cells appear to play a significant role in CAR-T cell induced neurotoxicity as reports have shown significant increases of CD14+ cells in patients with grade 3 or higher neurotoxicity^97^ and a pivotal large B cell lymphoma CAR-T cell clinical trial showed that among serum biomarkers associated with development with grade 3 or higher neurotoxicity, GM-CSF elevation was most significantly associated with neurotoxicity^3^. Recent preclinical studies have demonstrated that neurotoxicity and CRS are decreased and CAR-T cell activity is increased after inhibition of macrophage activating and monocyte activating cytokine GM-CSF with lenzilumab^87,98,99^. GM-CSF mutational inactivation also appears to have similar effects in CAR transduced T cells^98,100^.

Therefore, these findings suggest that GM-CSF neutralization helps diminish neurotoxicity and reduce CRS^98^. In addition, deletion of tyrosine hydroxylase in a myeloid cell specific manner or inhibition of this enzyme using metyrosine results in decreased catecholamine and cytokine levels^101^. Preclinical evidence also suggests that IL-1 receptor antagonists reduced a form of neuroinflammation in leukemia/lymphoma mouse models treated with CD19 targeted CARs^102^.

### CAR “off-switches”

Another potential avenue to ameliorate CAR-T cell toxicity is through implementing “off-switches” or suicide gene strategies. Such strategies would facilitate the ability to selectively decrease engineered cells at the onset of adverse events through the treatment with a secondary inducing agent^103^. Several approaches utilizing these concepts have been developed. For instance, independent expression or CAR constructs engineered to express full length CD20 or CD20 mimotopes facilitate the depletion of CAR-T cells via treatment with rituximab^104^. A limitation with this approach, however, is the relatively slow onset of antibody-mediated depletion of CAR-T cells may limit the efficacy of this approach in patients that require immediate reversal during severe, acute cytokine-mediated toxicities. This led to the impetus to develop faster switches such as inducible cas9, which in a clinical trial eliminated >90% of engineered T cells within 30 min^105^. Other strategies have relied on protease-based small molecule-assisted shutoff CARs (SMASh-CARs), which are also referred to as switch-off CARS (SWIFF-CARs)^106^. The biggest limitation with suicide strategies or other similar approaches is that although they are attractive for ensuring safety, their use abruptly stops therapy for rapidly progressing disease. This limitation has served as a strong incentive to develop strategies to ensure safety while leaving suicide gene activation as the last resort. One approach with exciting potential involves the use of dasatinib, a tyrosine kinase inhibitor, which functions to suppress the activation of T cells through inhibiting proximal TCR signaling kinases^107^. In preclinical models, dasatinib quickly and reversibly prevents the activation of CAR-T cells, and administration of dasatinib early after CAR-T cell infusion results in a significant mortality reduction of mice from otherwise fatal CRS^107^. Thus, this approach appears to provide temporary inhibition of CAR-T cell function and could allow for the rescue of CAR-T cell therapy after toxicities have subsided. In the future, the development of additional innovative approaches that temporarily inhibit CAR-T cell function and allow for CAR-T cell therapy rescue once the toxicity subsides will be necessary for CAR-T cell therapy to move towards first-line therapy for both hematological malignancy and solid tumors.

## Conclusions

CARs are modular synthetic receptors that consist of four main components: an extracellular target antigen-binding domain, a hinge region, a transmembrane domain, and one or more intracellular signaling domains. CAR-T cells have revolutionized the treatment of certain hematological malignancies. However, obstacles still remain, which were discussed in this review. Training a workforce to meet the demands of this complex and evolving field is challenging and requires innovative curriculum development^6^. Antigen selection is critical to CAR-T cell function. Tumor cells can downregulate antigens due to the selective pressure of the CAR-T cells. Even with appropriate antigen targeting, on-target off-tumor effects can occur and cause associated toxicity. In solid tumors, getting CAR-T cells to traffic to and infiltrate the tumor is a challenge. This obstacle can be compounded by the immunosuppressive microenvironment of malignancies. Effective treatment also runs the risk of CAR-T cell-associated toxicities such as CRS and neurotoxicity. However, while there are challenges, new strategies and potential solutions continue to evolve and may provide a path forward to more effective and safer future therapies.
